# Exploring structural phase transitions of ion crystals

**DOI:** 10.1038/srep21547

**Published:** 2016-02-11

**Authors:** L. L. Yan, W. Wan, L. Chen, F. Zhou, S. J. Gong, X. Tong, M. Feng

**Affiliations:** 1 State Key Laboratory of Magnetic Resonance and Atomic and Molecular Physics, Wuhan Institute of Physics and Mathematics, Chinese Academy of Sciences, Wuhan, 430071, China; 2 University of the Chinese Academy of Sciences, Beijing 100049, China

## Abstract

Phase transitions have been a research focus in many-body physics over past decades. Cold ions, under strong Coulomb repulsion, provide a repealing paradigm of exploring phase transitions in stable confinement by electromagnetic field. We demonstrate various conformations of up to sixteen laser-cooled ^40^Ca^+^ ion crystals in a home-built surface-electrode trap, where besides the usually mentioned structural phase transition from the linear to the zigzag, two additional phase transitions to more complicated two-dimensional configurations are identified. The experimental observation agrees well with the numerical simulation. Heating due to micromotion of the ions is analysed by comparison of the numerical simulation with the experimental observation. Our investigation implies very rich and complicated many-body behaviour in the trapped-ion systems and provides effective mechanism for further exploring quantum phase transitions and quantum information processing with ultracold trapped ions.

The laser-cooled ions confined in radio-frequency (rf) traps and Penning traps can condense into crystalline states and form different ordered configurations under control of the confining potentials^1^,^2^,^3^,^4^,^5^,^6^,^7^,^8^,^9^,^10^,^11^,^12^. The variation of the configurations corresponds to structural phase transitions of the ion crystals, such as the linear-to-zigzag phase transition predicted in theory^13^,^14^ and later experimentally verified^15^,^16^,^17^. Recent observations have also found that rapid implementation of the linear-to-zigzag phase transition leads to formation of defects in the ion crystal chain, obeying the inhomogeneous Kibble-Zurek mechanism, a topological phase transition relevant to the early universe^18^,^19^,^20^,^21^,^22^,^23^,^24^. Besides, if those ion crystals are laser-cooled down to ultracold states, it was predicted theoretically that the structural phase transition of the ion crystals from the linear to the zigzag can be mapped into an Ising model in a transverse field^25^,^26^,^27^. Different from in the classical regime, the linear-to-zigzag phase transition occurring in quantum regime is temperature dependent^28^. So the trapped ion crystals provide an experimental toolbox to explore the variation from a classical phase transition to a quantum counterpart^29^.

In the present work, we demonstrate the control of the ion crystals in our home-made surface-electrode trap (SET). The SETs have recently attracted much attention due to relatively simple fabrication as well as the possibility of trapping and shuttling short linear ion crystals, the latter of which is the prerequisite of a scalable quantum information processing^30^,^31^,^32^,^33^,^34^,^35^. Even in the case of a few ions, the confined ion crystals in a single layer lattice structure^36^,^37^, the controllable geometric structures of the ions and the flexible architecture of electrodes^31^,^32^ make the SETs very promising for quantum simulation, such as for the spin-spin coupling models^26^,^27^ in condensed matter physics and for fundamental feature in thermodynamics^38^,^39^. Particularly, the variable two-dimensional geometry of qubits in SETs is essential to measurement-based quantum computing^40^,^41^, error correcting codes^42^,^43^ and quantum annealing^44^,^45^.

Our present work intends to explore structural phase transitions with laser-cooled ^40^Ca^+^ ion crystals. The various conformations of the ions reflect typical many-body behaviour and also form the prerequisite of trapped-ion quantum information processing, from which we may know the distribution of the future qubits under our control and the influence from the rf heating. Due to strong asymmetry in our SET with the potential well in the *y*-axis much steeper than in both *x*- and *z*-axes, the ion crystals are distributed only in the *xz* surface (further justified later). As such, we define the anisotropy as 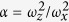, rather than 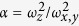
13,14,15,16,17, to characterize different ion crystal conformations, where 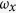 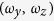 is the trap frequency in *x* (*y*, *z*)-axis. With up to sixteen laser-cooled ^40^Ca^+^ ion crystals, we will demonstrate different configurations in variation with *α*, and explore second-order phase transitions never experimentally identified before. By comparing simulated results with the experimental observation, we will also investigate the rf heating along different directions using the Langevin thermostat molecular dynamics (MD) method.

## Results

### Experimental setup and trapping potentials

**Figure 1:**
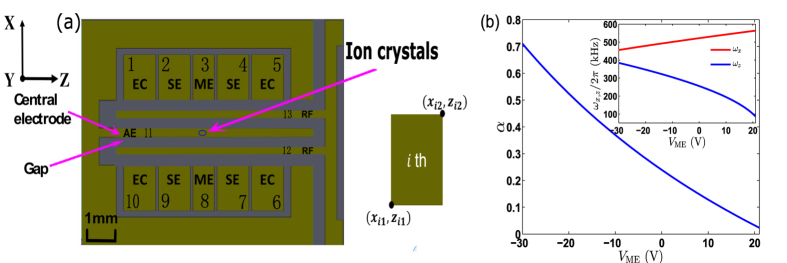
(**a**) Schematic of our SET in top view, consisting of a central electrode, two rf electrodes and two outer segmented dc electrodes, where the rf electrodes, the central electrode and the gaps in between are of the same width of 500 μm. Each outer segmented electrode consists of five component electrodes, i.e., a middle electrode, two control electrodes and two end electrodes. The widths of the control electrodes and end electrodes in the segment are 1.5 mm, and the middle electrode is 1 mm wide. The gap in the segmented electrodes is of 130 μm width. For understanding the potential of the SET, we label the electrodes from No. 1 to No. 13, and define 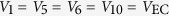, 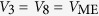, 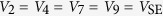, and 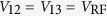. 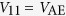 is for the potential compensation. The inset presents the definitions of 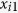, 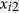, 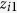 and 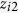 in Eq. (1) for the *i*th electrode. (**b**) The parameter 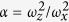 varying with the values of V_ME_ applied on the middle electrodes. Inset: the corresponding trap frequencies in variation with V_ME_. We have assumed a perfect compensation in this case (See details in Supplementary Information).

Our SET is a 500 μm scale planar trap with five electrodes^46^,^47^,^48^,^49^,^50^ as shown in Fig. 1(a). The electrodes are made of copper on a vacuum-compatible printed circuit board substrate. The electrodes labeled as EC and ME represent the end electrodes and middle electrodes, respectively, and SE represents four control electrodes. There are three horizontal electrodes as central electrodes, two of which, i.e., the RF electrodes, are applied by rf voltages and the middle one AE applied by a dc voltage works as a compensation electrode.

The radial electric potential 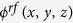 is produced by the rf voltage with amplitude V_RF_ ~ 640 V (0-Peak) and frequency 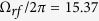 MHz. The axially dc electric potential 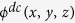 is produced by a voltage V_*EC*_ = 40 V applied on the end-cap electrodes. Depending on the parameters above, the rf potential null is above the trap surface by about 910 μm (See Supplementary Information), which does not generally coincide with the dc potential minimum. As a result, throughout the experimental implementation, we keep the two minima overlapping by adjusting the compensation voltage, which can effectively reduce the rf heating.

For convenience of description, we label the SET electrodes from 1 to 13, with the electrodes No. 1 to No. 11 applied by dc voltages, and the other two, i.e., No. 12 and No. 13, by rf voltages, as plotted in Fig. 1a. With dc voltages *V*_*i*_ applied on the *i*th electrode, static potential is generated as^51^


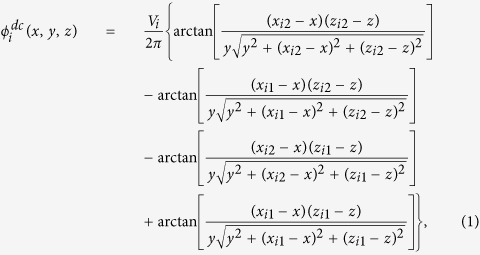


where 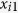, 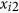, 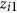 and 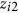 are labeled in the inset of Fig. 1a. For the two rf electrodes, we have 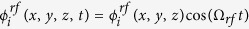 (*i* = 12, 13) with the rf frequency Ω_*rf*_. So the total effective trapping potential energy 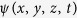 in the SET at time *t* is given by,


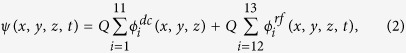


where *Q* is the ion charge. For clarity and simplicity, we employ the pseudo-potential approximation in part of our treatments below, where the pseudo-potential energy 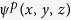 is expressed as


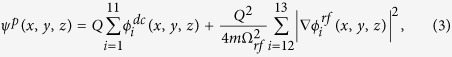


with *m* the ion mass. So for N ions confined in the SET, the total potential energy is given by 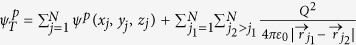


### Experimental observation and numerical simulation

Our experiment starts from the loading of ^40^Ca^+^ ions by two-stage photoionization^52^ using a 423 nm laser for the 4*S*_0_-4*P*_1_ transition of the calcium atoms, followed by the second excitation by a 380 nm light emitting diode. The trapped ions are Doppler cooled by a grating-stabilized 397 nm laser, with assistance of a grating-stabilized 866 nm laser for *D*_3/2_-state repumping^50^. We detect the 397 nm laser-induced fluorescence by an electron-multiplying CCD (EMCCD) (PhotonMax512, Princeton Instruments) along the *y*-axis. As a result, we cannot experimentally identify the ion crystals distributed along the *y*-axis. But our numerical simulation (See Methods and Supplementary Information) clearly identifies that, for the ions initially confined as a line in *z*-axis with 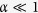, the ion crystals change to two-dimensional configurations in *xz* plane with the increase of *α*. Under the condition of *α* < 0.7, the ion crystals distribute for less than 2 μm along *y*-axis, 0 to 40 μm in *x*-axis and 50 to 150 μm in *z*-axis. As a result, there is no three-dimensional conformation of the ion crystals under current trapping condition.

**Figure 2:**
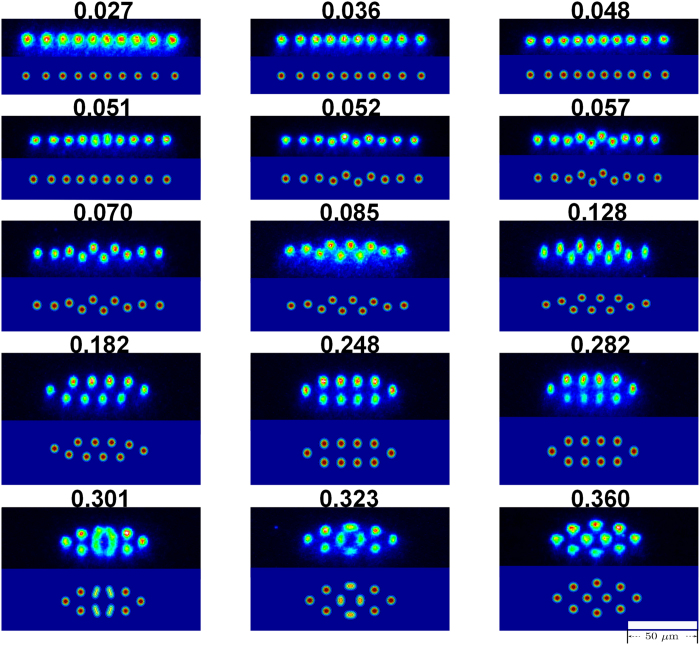
Crystals of ten ^40^Ca^+^ ions with different anisotropic values of the trapping potentials, with experimentally observed images (the upper of each panel) in comparison with numerically simulated results (the lower of each panel), where the horizontal direction means *z*-axis. The secular frequencies of the trapped ions are initially 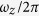 = 90 kHz, 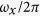 = 560 kHz and 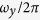 = 820 kHz. With increase of the axial confinement, a linear chain in 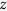-axis changes to a zigzag structure, and then to more complicated formations in the *xz* plane. Each image is labeled with a value of α. To demonstrate the key steps of the configuration changes, we choose some critical values of α. For the experimental observation with 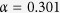, the blurring part in the image is due to a non-equilibrium state of the ions during the structural phase transition. Since the minimum energy analysis shows degeneracy (i.e., different configurations with the same energy) in this case, we overlap the degenerate solutions, which yields the elongated structure in the corresponding lower panel. There is a 50-μm scale bar in the bottom right-hand panel, which is drawn by considering the CCD resolution and 15 times magnification of the microscope objective before the CCD imaging. The scale bar applies to all the images.

For our purpose, we confine three to sixteen laser-cooled ions, for each of which we gradually raise or lower the trapping potentials and try to avoid hysteresis (or nonlinearity)^53^,^54^,^55^,^56^ in the observation of configuration changes of the ion crystals. In our operations, the voltage *V*_EC_ remains unchanged, but the voltage *V*_ME_ is applied on the middle electrodes decreased from 20 V to −30 V, which increases 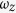 but decreases 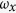, as shown in Fig. 1(b). So the ion crystal configuration changes with the increase of *α*. Meanwhile, the compensation voltage *V*_AE_ is adjusted to reduce the ions’ heating to the best, i.e., the dc potential minimum overlapping with the rf potential null. We plot the ion crystals involving ten ions as an example in Fig. 2 which images the change of the ion crystal spatial distributions for a wide range of the applied voltages on the middle electrodes. With the increase of *α*, the lower trapping frequency in *x*-axis leads to more serious rf heating (due to the ions more distant from the rf potential null) and the resolution blurring of the individual ions in our observation (see discussion later about heating). Some blurring cases, e.g. for 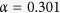, are also due to non-equilibrium states in the process of the structural phase transition. Nevertheless, considering the center of each ion, we may still identify the configurations of the ion crystals in those cases, which can be justified by the pseudo-potential approximation. As shown in Fig. 2, the observed configurations of the ion crystals are in good agreement with the simulated results under pseudo-potential approximation.

**Figure 3:**
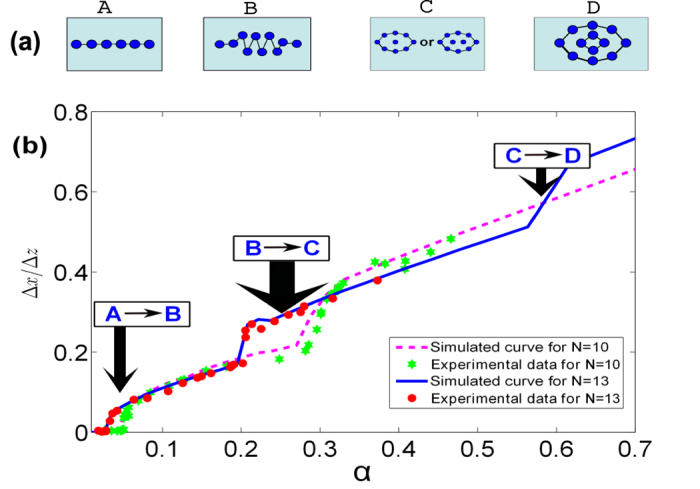
(**a**) Sketches of the ion crystal configurations; (**b**) Structural phase transitions of 10 and 13 ions with respect to the anisotropic parameter α of the trapping potential, where 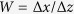 with 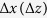 the center-to-center distance of two outermost ions in *x*(*z*)-axis. From the left to right, the abrupt raising occurs at the phase transitions from the linear to zigzag (*α* < 0.15), from the zigzag to the ellipse encircling a single ion or an ion string (0.2 < *α* < 0.3), and then to more complicated configurations with the concentric ellipses (*α* > 0.5), respectively. The simulation curves are plotted based on 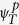.

To characterize the configuration changes, we employ the center-to-center distance Δ*x* (Δ*z*) of two outermost ions in 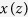-axis, which are found to be very sensitive to the potential change. By measuring Δ*x* and Δ*z* in each image of the ion crystal configuration, we define 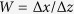 and find some abrupt raising in the curves of *W* with respect to *α*, implying the structural phase transitions. As shown below, *W* can be considered as an order parameter, which changes from zero to different non-zero values corresponding to different structural phases. Figure 3 exemplifies the cases of 10 and 13 ions with three such phase transitions in the change of the ion crystal configurations. With respect to the cusp-like phase transition in the thermodynamical limit, finite numbers of the ions only show the abrupt raising with definite slopes around the critical points of the phase transitions, in which the slopes vary for different numbers of the ions. The first phase transition, occurring at *α* < 0.15, is for the linear-to-zigzag phase transition which has been investigated previously in different ion-trap systems^13^,^14^,^15^. But the second one has never been reported experimentally before, which happens at 0.2 < *α* < 0.3, corresponding to the phase transition from the zigzag to the ellipse encircling a single ion or an ion string. The third phase transition represents the configuration change to a more complicated case, e.g., the concentric ellipses. Such a case, however, with *α* > 0.5, occurs in a much lower depth trap in *x*-axis, in which the ion crystal melting has handicapped our exact measurement. So the third phase transition in Fig. 3 is only theoretically predicted. We will come back to this point later by treating the rf heating in the case of *α* > 0.5.

### Power laws

To give a more complete impression on this topic, we list in Table 1 different structural phase transitions occurring for different numbers of the ions, where the few-ion cases (*N* ≤ 5) are omitted due to the same as the well-known results in previous publications^15^,^17^. Although our SET is different from the rf linear traps or Penning traps in the potential or the potential symmetry, there is no fundamental difference in ion crystal configurations if the ion number *N* is less than 6, where the only phase transition is from the linear to the zigzag. For more than five ions involved in the trapped ion crystals, however, there are more complicated configurations and thereby more phase transitions. This can be understood from Table 1 that more ions involved lead to more complicated configurations. Particularly, more phase transitions occur gradually with more ions involved, in which the same phase transition might occur in the case of a smaller *α*.

Table 1 also presents the possibility of experimentally observing the third phase transition with *α* < 0.5 if fourteen or more ions are involved in the ion crystals. However, our experiments with fourteen to sixteen ions show serious melting before reaching the critical point of the third phase transition. To understand the experimental difficulty, we have to consider the influence from the rf heating, as discussed later.

**Figure 4:**
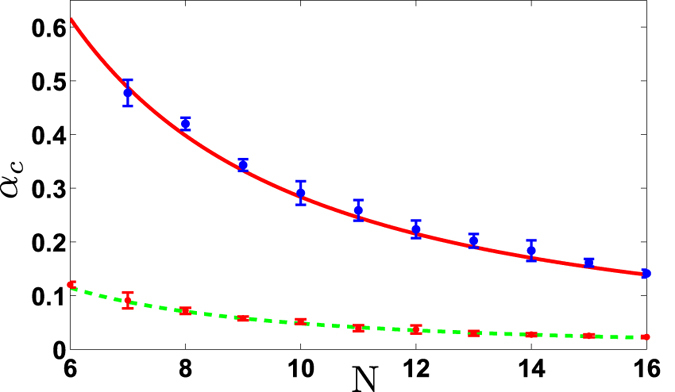
Power-law scalings of 6–16 ions with the measured and simulated values of the critical anisotropic parameters versus the ion number *N*, where the curves are plotted based on the calculation by 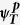 and the experimental data are averaged over the measured data around the critical points of the phase transitions. The lower (upper) curve is for the first (second) phase transition plotted in Fig. 3(b). The error bars of the experimental data are determined by the mean square root, as explained in the text.

The previous studies have shown the scaling behaviour at the critical point of the phase transition from the linear to the zigzag^13^,^14^,^15^,^17^. Here we assume the similar scaling behaviour in other phase transitions by defining 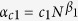 and 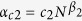, where 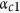 and 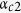 are, respectively, the critical anisotropic parameters for the first and second phase transitions, *N* is the ion number and 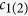 and 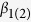 are the corresponding constants determined by the fitting. The third phase transition is not considered here due to lack of enough experimental data. We have compared the experimental data with the simulation values at the critical points of the two phase transitions in Fig. 4, where the curves are plotted by numerical simulation based on the definitions of the scaling behaviour given above and the experimental data are averaged from the observed data within the abruptly raising regimes of the curves in Fig. 3. We label in Fig. 4 the deviation from the average values of the measurements by error bars, which are determined by the mean square root. We find that the numerical values (i.e., the curves) fit the experimental data within the range of the statistical error.

## Discussion

Our experimental values above for the first phase transition are in very good agreement with the previous theoretical results^13^, even better than the results in^15^, as listed in Table 2. This might be due to the fact that the power-law expression intrinsically depends on the number of the ions involved^13^,^14^,^15^. We are working on 6–16 ions, more than considered in^15^, and thereby obtain the parameter values closer to that in Schiffer’s calculation (involving 10–500 ions) in^13^. Moreover, despite non-generality, the expression of the power law implies the onset of a second-order phase transition. Besides, the power-law expression is also useful for understanding the relevance of the phase transitions to the values of *α* in Table 1. Rewriting the power-law expression 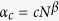 as 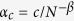, for the positive constant *c* and the negative constant *β*, we surely have smaller values of 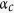 with more ions involved.

**Figure 5:**
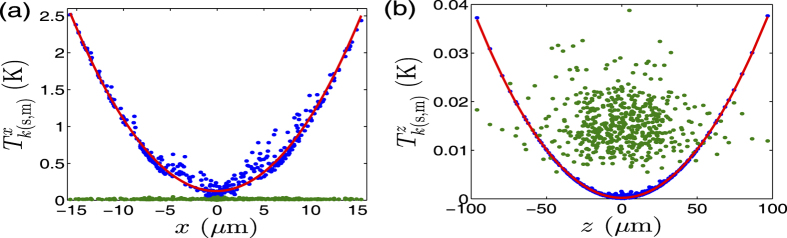
The ions’ temperature 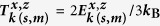, where the subscripts *s* and *m* mean contribution from the secular motion and the micromotion, respectively, and the superscripts *x* and *z* associate with *x*- and *z*-direction. The contribution from the micromotion (the secular motion), denoted by the blue (green) dots, is simulated by the MD method. For clarity, we fit the blue dots by red solid curves using quadratic functions.

Although our slow operations can be reasonably described under the pseudo-potential approximation, a complete consideration of the time-dependent potential in the SET is necessary for fully understanding the details in the configuration change of the ions, such as the ions’ heating due to the rf potential^57^,^58^. As such, we simulate the dynamics of the system by solving the MD equations (See Methods). The heating effect due to the micromotion of the ions occurs in three dimensions of the SET, which is strongly relevant to the positions of the ions from the rf potential null. In the case of few tens of trapped ions with *α* < 0.7, since our simulation identifies a tiny distribution of the ions along the y-axis and we constantly keep the potential minimum at the rf potential null by adjusting *V*_AE_, we may focus our investigation on the heating in *x*- and *z*-axes during the configuration change. The temperature of the ions is assessed by the kinetic energy owned by the ions. For a comparison, we compute the energies from both the secular motion and the micromotion in the two dimensions. As shown in Fig. 5, the micromotion energies in both directions are proportional to the distance square, behaving as quadratic functions. In contrast, the secular motion energies are near constants along *x*- and *y*- axes. Besides, the overall temperature in *z-*axis is much less than in *x*-axis, implying negligible heating in *z*-axis compared to in *x*-axis. This reflects a fact that we have negligible rf potential along *z-*axis (See Supplementary Figure 2). With the increase of *α*, the ion crystals form the configurations with more components away from *z*-axis, which leads to a rapid increase of the rf heating. Meanwhile, the increase of *α* means stronger rf heating and weaker confinement in *x*-axis. This is why we cannot observe experimentally the third phase transition in Fig. 3 since the ion crystals turn to be seriously melting when *α* > 0.5 and then escape from the trap in the case of a bigger *α*. More details for quantitative estimate of the energies can be found in Supplementary Information.

On the other hand, the discrepancy between the experimental values and the simulated results also indicates the imperfection in our operations with respect to the ideal consideration. We estimate the imperfection-induced errors within 4.7% and 7.5%, respectively, in the first and second phase transitions, including 0.15% error relevant to ±0.3 kHz deviation in measuring *x*- and *z*-axial frequencies, 0.03% (0.06%) error due to ±53 Hz (±166 Hz) uncertainty of the dc potential in *z*-(*x*-) axis and 4.45% error from ±13.34 kHz uncertainty in the *x*-axial rf potential. There are some other unclear errors in the second phase transition.

Following on from this work, we expect to explore quantum mechanically structural phase transitions, which can be mapped into a quantum phase transition of Ising model subject to a transverse field^25^,^27^ and demonstrate temperature dependence^28^, in future experiments by further cooling the ions down to the vibrational ground state. To this end, an improved SET with higher symmetric structure and deeper potential is expected. This new SET will also help implementing quantum computing tasks with ultracold ions confined and moved in a scalable fashion and error correcting codes and quantum algorithms accomplished under control. Particularly, the micromotion-induced heating might be effectively suppressed in the SET if the transverse motional modes and well-designed strong laser pulses are employed^59^.

## Methods

### The minimum energy analysis

**Figure 6:**
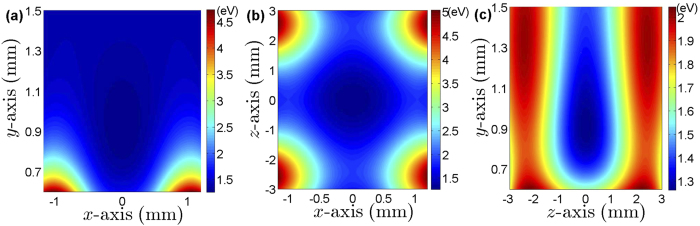
Simulation of the pseudo-potentials in the SET, where (**a**) is for the potential in *xy* plane at *z* = 0 mm, (**b**) is for the potential in *xz* plane at *y* = 0.91 mm, (**c**) is for the potential in *yz* plane at *x* = 0 mm. The parameters take the values of *V*_EC_ = 40 V, *V*_ME_ = −5 V, *V*_AE_ = 2.96 V, *V*_SE_ = 0 V and *V*_RF_ = 640 V.

Under the pseudo-potential approximation, the trapping potential of our SET can be analytically expressed as in Eq. 3 and plotted in Fig. 6. With the pseudo-potential, we have simulated the stable configurations of the ion crystals as in Fig. 2, where different trapping potentials induce different configurations and the position of each cooled ion can be solved by minimizing the total potential energy 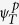. The energy minimum analysis is carried out by the gradient descent method^60^, i.e., a first-order optimization algorithm for finding a local minimum of a function.

### The numerical simulation involving the micromotion

A complete description of motion of the ion crystals requires involvement of the rf potential. In this case, the dynamics of the ions at a specific temperature *T* can be simulated by the MD method. For the *j*th ion, the Langevin equation is


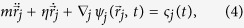


where 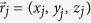 is the coordinate under the total energy potential 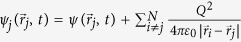, *m* is the mass of the *j*th ion and *η* is the friction coefficient induced by the laser cooling. 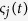 is the stochastic force which obeys following ensemble average relations: 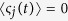 and 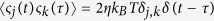. Eq. (4) is numerically simulated using the values 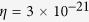 kgs^−1^ and 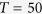 mK by the Brownian dynamics^18^,^19^,^61^.

## Additional Information

**How to cite this article**: Yan, L. L. *et al.* Exploring structural phase transitions of ion crystals. *Sci. Rep.* **6**, 21547; doi: 10.1038/srep21547 (2016).

## Supplementary Material

Supplementary Information

## Figures and Tables

**Table 1:** List of the structural phase transitions happening within different regimes of *α* for 6–16 ions, where 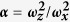, and different configurations are sketched in Fig. 3(a): A, Line; B, Zigzag; C, Ellipse with a single ion or a line of ions encircled; D, Concentric ellipses.

*N*	6	7	8	9	10	11	12	13	14	15	16
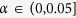						A → B	A → B	A → B	A → B	A → B	A → B
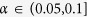		A → B	A → B	A → B	A → B						
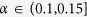	A → B									B → C	B → C
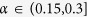						B → C	B → C	B → C	B → C		
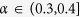				B → C	B → C						
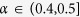		B → C	B → C						C → D	C → D	C → D
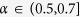	B → C							C → D			

We call A→B, B→C and C→D the first, second and third phase transitions, respectively.

**Table 2:** Scaling constants from fits of experimental data and simulated results in Fig. 4 in comparison with previous results^13^,^15^, where the uncertainties in both the numerical values and the experimental data are standard errors in our linear regressions with 95% degrees of confidence.

	OurExperiment	OurSimulation	Resultsin^13^	Results in^15^
*c*_1_	2.53 ± 0.45	2.41 ± 0.13	2.53	2.94 ± 0.07
*β*_1_	−1.71 ± 0.08	−1.70 ± 0.02	−1.73	−1.80 ± 0.01
*c*_2_	8.65 ± 1.35	9.36 ± 1.32		
*β*_2_	−1.47 ± 0.07	−1.52 ± 0.06		

Only the first phase transition was considered in^13^,^15^.
