# Supplementary material for: Exploring structural phase transitions of ion crystals
Source: Sci Rep. 2016 Feb 11;6:21547. doi: 10.1038/srep21547 (PMC4749997; doi:10.1038/srep21547)
Supplement: Supplementary Information [file srep21547-s1.pdf]

Supplementary information for exploring structural phase transitions of ion crystals

L. L. Yan[1,2], W. Wan[1,2], L. Chen[1,*], F. Zhou[1,†], S. J. Gong[1,2], X. Tong[1], and M. Feng[1,‡]

[1]State Key Laboratory of Magnetic Resonance and Atomic and Molecular Physics, Wuhan Institute of Physics and Mathematics, Chinese Academy of Sciences, Wuhan, 430071, China
[2]University of the Chinese Academy of Sciences, Beijing 100049, China
[*]liangchen@wipm.ac.cn
[†]zhoufei@wipm.ac.cn
[‡]mangfeng@wipm.ac.cn

ABSTRACT

We discuss here the details of the trapping potentials, the kinetic energies and the rf heating in three dimensions of the trap.

Trapping potentials of the surface electrode trap

Under the pseudo-potential approximation, the potentials of the surface electrode trap (SET) in three dimensions vary with the voltage $V_{\rm ME}$ applied on the ME electrodes (see Supplementary Figure 1(ax-az)). We consider the case of a perfect compensation, implying the pseudo-potential minimum exactly at the rf potential null. Since the ion crystals are trapped within a small region around the pseudo-potential minimum, i.e., the trap center (x_0, y_0, z_0), the trapping pseudo-potential can be approximated by power series expansions,[1]

$$\psi^p(x,y,z) = \sum_{k,l,m} C_{k,lm}(x-x_0)^k(y-y_0)^l(z-z_0)^m, \tag{1}$$

where $C_{k,lm}$ are coefficients to be determined later by comparing with Eq. (3) in the main text. As a good approximation, we may assume the pseudo-potentials in the vicinity of the trap center as harmonic oscillator potentials: $\psi^p(x,y_0,z_0) = \frac{1}{2}m\omega_x^2x^2$, $\psi^p(x_0,y,z_0) = \frac{1}{2}m\omega_y^2(y-y_0)^2$ and $\psi^p(x_0,y_0,z) = \frac{1}{2}m\omega_z^2z^2$ with $x_0 = z_0 = 0$ and y_0 =910 μm. As such, the trapping frequencies in three dimensions can be obtained, as in Supplementary Figure 1(b).

To make sure the the pseudo-potential minimum overlapping exactly with the rf potential null, we have to keep adjusting the compensation voltage $V_{\rm AE}$ in the variation of $V_{\rm ME}$. By simulation, we find that this compensation can be accomplished based on a linear relation between $V_{\rm ME}$ and $V_{\rm AE}$, as plotted in Supplementary Figure 1(c)).

Kinetic energies of the secular motion and the micromotion

In order to estimate the heating effect quantitatively, we calculate the ions' temperature in three dimensions by the relation $T^{x,y,z}_{\rm s,m} = 2E^{x,y,z}_{k(\rm s,m)}/3k_{\rm B}$ with the subscripts s and m denoting the secular motion and the micromotion, $E^{x,y,z}_{k(\rm s,m)}$ the energy distributions along $x-$, $y-$ and $z-$axes, and $k_{\rm B}$ the Bolztmann constant. The root-mean-square (rms) velocity for the ith ion is defined as $(v^{\alpha}_{{\rm rms},i})^2 = \sum_{j=1}^{J}(v^{\alpha}_{i,j})^2/J$, where $\alpha = x,y,z$, j and J denote the jth time step and the total time steps in one rf period. The secular velocity equals the average velocity over one rf period $\bar{v}^{\alpha}_i = \sum_{j=1}^{J} v^{\alpha}_{i,j}/J$.[2] As a result, the temperature regarding the secular motion is calculated as $T^{\alpha}_{{\rm s},i} = 2E^{\alpha}_{k({\rm s}),i}/3k_{\rm B} = \frac{m}{3k_{\rm B}}\langle(\bar{v}^{\alpha}_i)^2\rangle$ with $\langle\cdots\rangle$ the average over many rf periods, and the total kinetic energies for the ith ion can be calculated by $E^{\alpha}_{k,i} = \frac{1}{2}m\langle(v^{\alpha}_{{\rm rms},i})^2\rangle$. Therefore, the temperature due to the micromotion is $T^{\alpha}_{{\rm m},i} = 2E^{\alpha}_{k({\rm m}),i}/3k_{\rm B} = \frac{m}{3k_{\rm B}}[\langle(v^{\alpha}_{{\rm rms},i})^2\rangle - \langle(\bar{v}^{\alpha}_i)^2\rangle]$.

Heating is resulted from the micromotion of the ions. So we focus below on the rf potentials in the SET. Specific calculation shows that the rf potential in $z-$axis remains nearly constant as low as the rf potential null. As plotted in Supplementary Figure 2(a), the rf potential along $z-$axis is nearly negligible compared to the counterpart along x-axis. We also calculate the temperature variation along the y-axis in Supplementary Figure 2(b), which helps to understand the minimum potential (at 910 μm away from the trap surface) in the direction unavailable for experimental observation. Due to the compensation making the potential minimum overlapping with the rf potential null, the motion of the ion crystals along y-axis is restricted within a very small regime (about 1 μm) and thus the micromotion energy in the y-direction is small and comparable to the secular motion energy. This implies that the ion crystals only vary two dimensionally in the xz-plane and thus we only need to consider the heating from x-axis.

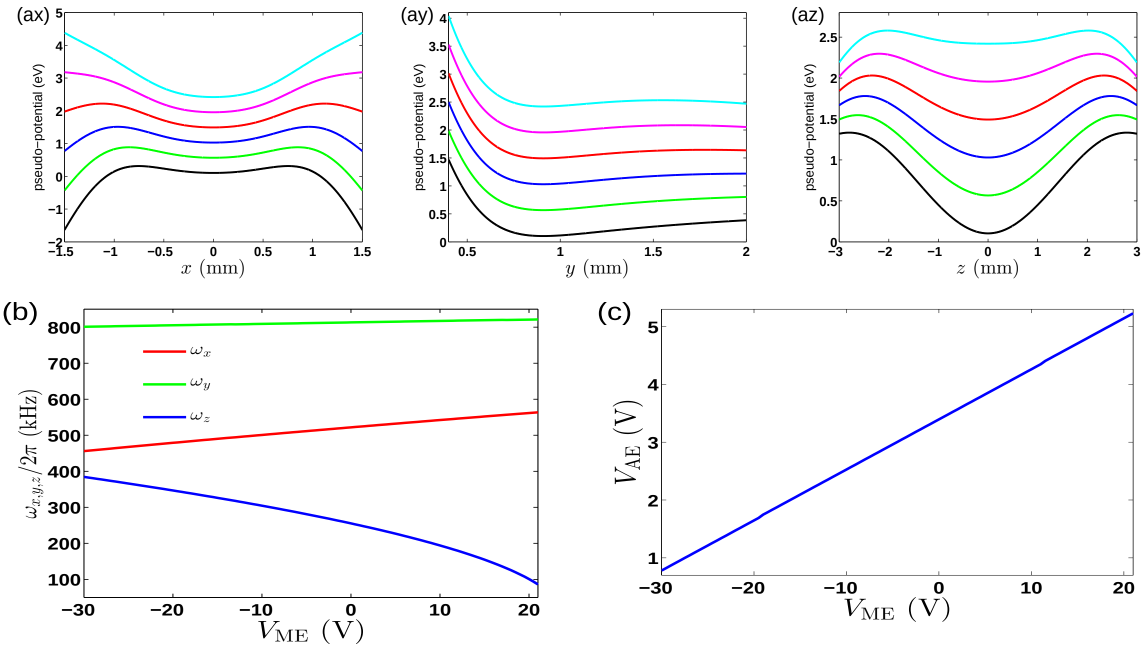

Figure 1. (ax-az) The pseudo-potential distributions along x-, y- and z-axes. The curves from the bottom to top denote the cases with voltages applied on the ME electrodes $V_{\mathrm{ME}} = -30, -20, -10, 0, 10, 20$ V. (b) The three-dimensional trap frequencies in the vicinity of the pseudo-potential center in variation with $\mathrm{V_{ME}}$ applied on the middle electrodes. (c) The linear relation between the operation voltage V_{ME} and the compensation voltage V_{AE}.

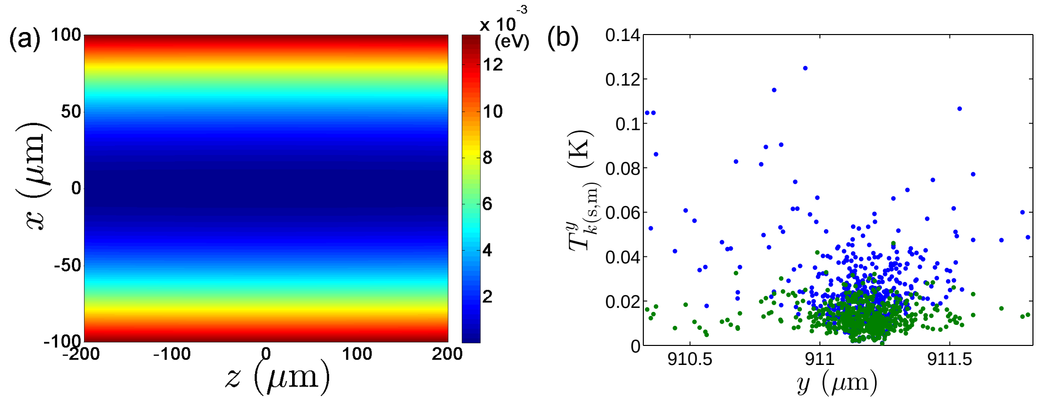

Figure 2. (a) Distribution of the rf potential in the xz-plane. (b) The ions' temperature $T^{y}_{k(\mathrm{s,m})}$ distributed along the y-direction, where the blue (green) dots denote the temperature due to the micromotion (the secular motion).

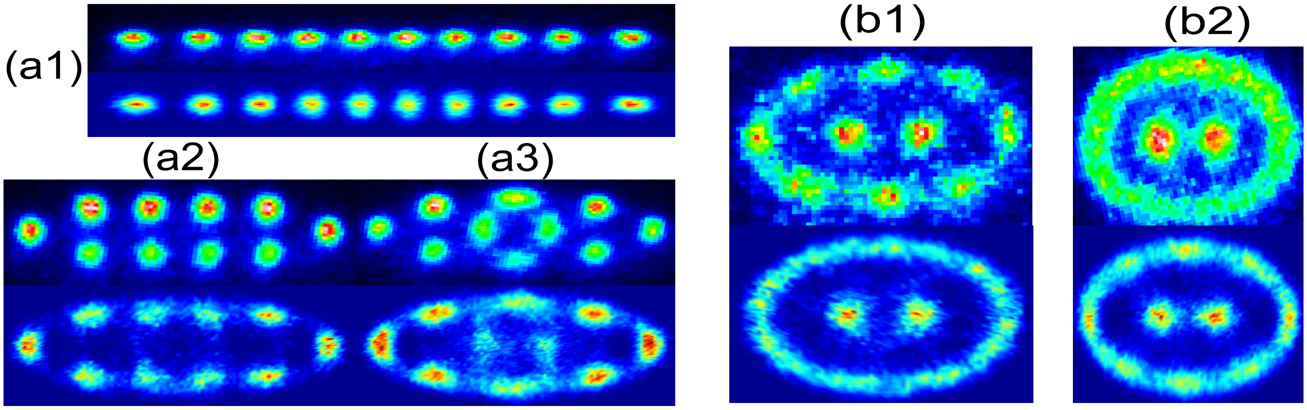

Figure 3. Crystals of ten $^{40}Ca^{+}$ ions with different anisotropic values of the trapping potentials, with experimentally observed images (the upper of each panel) in comparison with the MD simulation (the lower of each panel), where the horizontal direction means z-axis. (a1) α =0.036, (a2) α =0.248, (a3) α =0.323, (b1) α =0.524 and (b2) α =0.820.

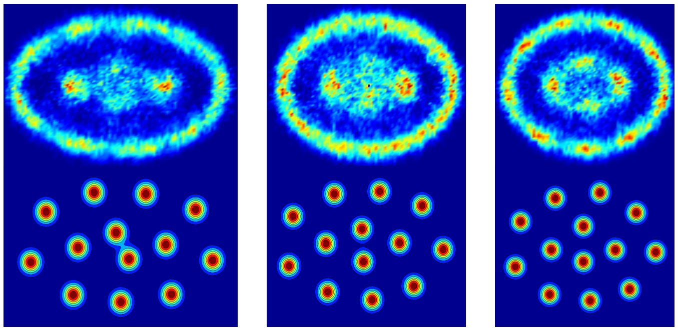

Figure 4. Configurations of thirteen ions in crystals by the MD simulation (upper image in each panel) with α =0.524 (Left panel), 0.613 (Middle panel) and 0.711 (Right panel), respectively. As a comparison, we also present the pseudo-potential simulation results in the lower images of the panels.

Simulation for heating and melting

Employing the Langevin thermostat molecular dynamics (MD) method, we simulate again the configurations of ten ions in Supplementary Figure 3. Compared with the pseudo-potential simulation in Fig. 2 of the main text, the MD simulations demonstrate characteristics of the ion crystals more accurately, including the heating effects. With the increment of α, heating becomes more serious, and it is hard to clearly distinguish individual crystals when α >0.5.

Correspondingly, we also simulate the cases with thirteen ions as plotted in Supplementary Figure 4, in which more complicated configurations are presented and meanwhile heating is more serious than the cases of ten ions. Since no experimental data is available in these cases, we compare the results of the MD results with those of the pseudo-potential simulation. With respect to the false-color images by the pseudo-potential simulation, the MD simulation can image strong influence from the rf heating that the ions in outer layer turn to be indistinguishable from each other.

Other observations for the second structural phase transition

We carry out the experiments for the structural phase transition with different ions. Besides the ten-ion cases demonstrated in Fig. 2 of the main text, we have also investigated other cases by comparing observations with the pseudo-potential simulations. Here we present some of these results in Supplementary Figure 5, mainly displaying the changes in the vicinity of the critical points of the second structural phase transitions for different ion numbers. We have to mention that we only present the pseudo-potential simulation here. A MD simulation involving the micromotion might partially explain the blurring images in Supplementary Figure 5, and the blurring parts are also partially resulted from the non-equilibrium states during the structural phase transitions. For example, for the case of $N = 11$, the equilibrium configuration exists at α =0.23 and 0.357. With the increase of α from 0.23, before reaching 0.357, the configuration is non-equilibrium, which is reflected in simulation as the solution degeneracy, i.e., different configurations with the same energy. As such, the corresponding panels are drawn with the overlapped degenerate solutions. Further serious exploration of the melting would be definitely helpful for understanding our

SET under a realistic condition.

References

1. Mokhberi A. and Willitsch S. Structural and energetic properties of molecular Coulomb crystals in a surface-electrode ion trap. *New J. Phys.* **17**, 045008 (2015).
2. Zhang, C. B., Offenberg, D., Roth, B., Wilson M. A. and Schiller S. Molecular-dynamics simulations of cold single-species and multispecies ion ensembles in a linear Paul trap. *Phys. Rev. A* **76**, 012719 (2007).

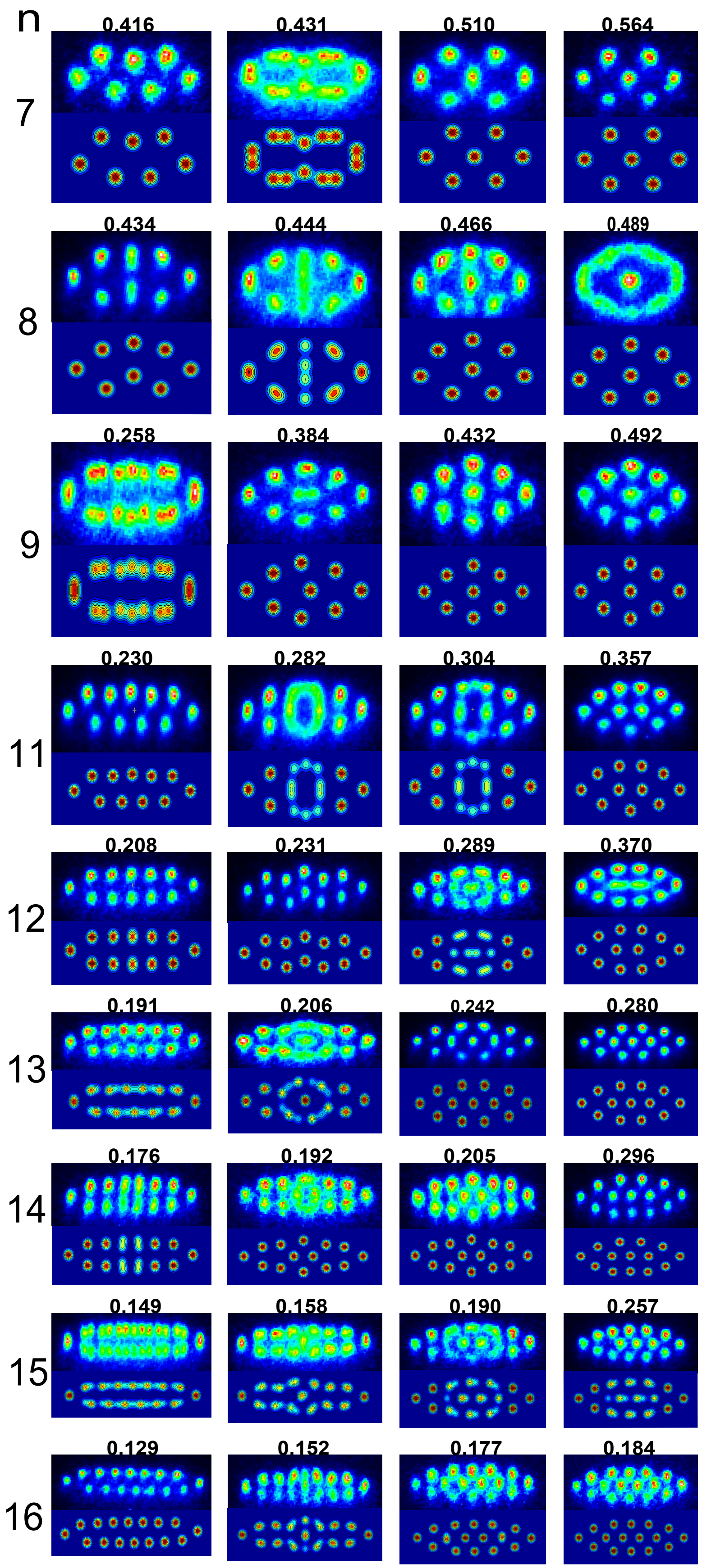

Figure 5. Configurations of seven∼sixteen ions crystals in the vicinity of their corresponding critical points. In each panel, the upper (lower) denotes the experimental observation (the pseudo-potential simulation).
